# Hypocrisy Around Medical Patient Data: Issues of Access for Biomedical Research, Data Quality, Usefulness for the Purpose and Omics Data as Game Changer

**DOI:** 10.1007/s41649-019-00085-3

**Published:** 2019-06-01

**Authors:** Erwin Tantoso, Wing-Cheong Wong, Wei Hong Tay, Joanne Lee, Swati Sinha, Birgit Eisenhaber, Frank Eisenhaber

**Affiliations:** 1 Bioinformatics Institute (BII), Agency for Science and Technology (A*STAR), 30 Biopolis Street, #07-01, Matrix, Singapore, 138671 Singapore; 2 School of Computer Science and Engineering (SCSE), Nanyang Technological University (NTU), 50 Nanyang Drive, Singapore, 637553 Singapore

**Keywords:** Patient data privacy, Clinical patient data quality, Electronic health record, Omics data, Genome sequencing

## Abstract

Whether due to simplicity or hypocrisy, the question of access to patient data for biomedical research is widely seen in the public discourse only from the angle of patient privacy. At the same time, the desire to live and to live without disability is of much higher value to the patients. This goal can only be achieved by extracting research insight from patient data in addition to working on model organisms, something that is well understood by many patients. Yet, most biomedical researchers working outside of clinics and hospitals are denied access to patient records when, at the same time, clinicians who guard the patient data are not optimally prepared for the data’s analysis. Medical data collection is a time- and cost-intensive process that is most of all tedious, with few elements of intellectual and emotional satisfaction on its own. In this process, clinicians and bioinformaticians, each group with their own interests, have to join forces with the goal to generate medical data sets both from clinical trials and from routinely collected electronic health records that are, as much as possible, free from errors and obvious inconsistencies. The data cleansing effort as we have learned during curation of Singaporean clinical trial data is not a trivial task. The introduction of omics and sophisticated imaging modalities into clinical practice that are only partially interpreted in terms of diagnosis and therapy with today’s level of knowledge warrant the creation of clinical databases with full patient history. This opens up opportunities for re-analyses and cross-trial studies at future time points with more sophisticated analyses of the same data, the collection of which is very expensive.

## Introduction

When a non-clinical researcher wants to test a hypothesis or study trends with medical data for the first time, the access to the data is typically barred due to arguments of patient data privacy. We speak out of own experience as it took many years for the Bioinformatics Institute Singapore to get inroads into medical data analytics. Our first entry into the field finally happened by accident when a large academic clinical trial in Singapore under the threat of failure urgently required professional data handling and analysis support for rescue.

The privacy argument is both simplistic and hypocritical as we will see below. It is simplistic as it takes much more than the patients’ consent for medical records to become a tool in biomedical research. For example, the profane side of it is that medical records in their original form are often unusable for research due to many inaccuracies and outright errors. Examples will be provided below in the text. This technical aspect has an immediate ethical dimension as it might prompt fruitless avenues of research or even generate apparent ‘scientific’ arguments for justifying useless or dangerous therapies.

Bringing the privacy argument into the forefront of public discussion is also hypocritical as is allows hiding complexities of extracting benefits from medical records from the public eye. The role of other stakeholders is not nearly as intensively discussed. The access to medical patient data is in the center of a conflict between the vested interests of various groups: (i) the patients who do not want their data being misused but who are generally happy to trade in privacy for the prospects for better biomedical science and improved therapy, (ii) the clinicians who collect and guard the medical data and who might fear that others consider the data or its analysis messy, (iii) the bioinformaticians who cannot move forward scientifically without the data. There is also the corporate interest of healthcare providers who consider access to patient data as part of their intellectual property and who monetize it by optimizing their own services or via clinical trial contracts with industry. Thus, clinical groups and healthcare providers have an interest (and the means) to restrict outside access to their clinical data for economic and academic reasons; yet, these are arguments that are difficult to be upheld in public. Finally, there are the administrators who wish to prevent scandals of data misuse. The conflict would be easier to resolve if it was not between contradicting short-term interests of various stakeholders and the great but fuzzy promise of improved and lower-cost medicine in the longer term. The patients’ point is simply easier to defend publicly; so, this aspect gets hypocritically over-represented in the public discourse, conveniently overshadowing interests of other parties that are not so easy to justify.

## The Issue of Patients’ Data Access in the Era of Omics Technologies Entering Healthcare

The conflicts between these different interests are getting more acute recently as a result of (i) cost pressures in health care and in biomedical research, (ii) digitalization of health care, and (iii) the wide-spread availability and introduction of omics technologies. The health care sector is one of the prime targets for the digitalization revolution happening in front of our eyes. The reasons are manifold and include aspects of evidence-based medical therapy, increasing amount of clinical, laboratory, imaging, and especially omics data collected per patient over the time of observation, the need of their analysis, the complexity of treatment options, legalities and documentation issues, etc. Yet, the process is primarily driven by the need to provide services of increasing sophistication to an ever larger cohort of patients (that ultimately encompasses all population living on a given territory) for affordable prices. At the same time, the cost per service act is societally required to go down and the overall increase of health care costs is expected to be dampened. Most of all, computerized support cuts down the working time of healthcare personnel needed, allows the provision of teleservices for patients at home, and opens new opportunities to control and optimize resource consumption in the system.

As a byproduct, large electronic databases of medical patient data are emerging (Electronic Health Records, or EHRs) that offer new, unprecedented opportunities for biomedical research. Whereas direct biological experimentation on humans (as with model organisms in the laboratory) is severely restricted by ethics, law and, most often, is outright impossible, the histories of diagnoses, therapies, and outcomes are recorded for huge numbers of real patients as a result of their medical care. Though EHRs were unintended for research, the data recorded allow quantitatively assessing correct medical decision making and efficiency of treatment as well as doing basic research on physiological mechanisms and pathological reactions.

A completely new dimension is added by omics data recording coming into the hospital setting. Genomic aberrations can be related to medical histories now. In principle, medical records with omics data are what is called in experimental biology “deep phenotyping with long time series”, the most informative of all data. In the long run, efficiency of treatment can be improved (and, ultimately, costs could be brought down) by adapting interventions to the genetic makeup and variants of biomolecular processes of specific patient groups or even individuals (what is sometimes called personalized medicine). In addition to individual gains, society will benefit due to the extension of health span and of duration of work life, decreasing costs for care and cure, etc.

Medical institutions might become the main source of data for studying genome-phenotype relationships that will outclass traditional biological laboratories in the future. This process is irreversible and will be deepened in the time to come. New, cheap technologies in high-throughput data generation, especially for biomolecular sequencing, are in the process of making genome sequencing, mRNA, non-coding RNA, protein and cellular profiling increasingly affordable for application over ever wider groups of patients. In parallel, new imaging modalities both at the level of organ and full body imaging as well as for the in situ visualization of molecular processes at tissue level penetrate into medical practice. Further unprecedented opportunities arrive with the application of physiology monitoring devices and companion software applications capable of generating alerts and sharing data with health care providers or social networks (Lim et al. 2018; Shameer et al. 2017).

The ability to interpret the patients’ medical data for the purpose of diagnostics and treatment is very limited with today’s level of knowledge in life sciences. At the same time, collecting this data just for one specific research project is costly and its re-collection in the future from the same patients, maybe, is impossible. Therefore, it makes great sense to store these data in databases for long term and to reanalyze the same records with more sophisticated concepts and formalized approaches in a myriad of future research projects. At a later time point when research methodology has further evolved, research breakthroughs might become possible with essentially zero additional data collection costs. This is especially true for new data types coming from image modality innovations or recently developed omics technologies. For example, less than 1% of the human genome sequence has at least some biological interpretation and that status will remain unchanged for years and decades to come if the pace of research does not change compared with the recent past (Eisenhaber 2012; Kuznetsov et al. 2013; Sinha et al. 2018).

In the light of these considerations, one might believe that analyzing various patients’ data sets is the most normal thing in biomedical and health care research today. Yet, the reality cannot be more different as we will see below. This article is based on own experiences in attempting medical data analysis at the Bioinformatics Institute Singapore as well as on conversations with stakeholders in Singapore and abroad over many years. We will first consider various hindrances that limit the access of life science research to medical patient data. Thereafter, our attention will shift towards the topic of quality of various types of patient data that essentially determines its usefulness for these scientific purposes. We will also discuss possible solutions of some of these complicated problems and we will see how technical, medical, and bioethical aspects in the matter of patient data studies interact with, support and limit each other.

## Access to Medical Patient Data in Biomedical Research: the Patients’ View in the Matter of Data Privacy Versus Medical Innovation

The right of patients to keep their medical data confidential is part of the basic human right for the protection of privacy and explicitly regulated by law; for example, the Personal Data Protection Act (PDPA) in Singapore (Personal Data Protection Commission 2012) protects personal data while providing regulations and imposing restrictions for organizations and individuals that use these data. Indeed, ethical considerations implemented in professional standards dating back to the oath of Hippocrates and enhanced by PDPA-type law and punishment mechanisms prohibit the use of medical data in ways that might result in damaging the patients’ individual and group interests.

Yet, there is a hierarchy of values and, in fact, the right for data privacy is not even at the top of many patients’ agenda. The right for patient data privacy clashes with the more basic need of patients to live and to prevent death and disability, to receive efficient medical treatment for their condition when the costs for state-of-the-art treatment are exorbitant or, especially, when the current level of medical science cannot deliver effective care even at the symptomatic level due to the limit of knowledge and insight into pathogenetic mechanisms. To emphasize, modern medicine does not deliver desired outcomes for a wide range of conditions.

Thus, patients’ data privacy and the need for medical innovation conflict. Without access to patient data for research, progress in the medical sphere is impossible. Many patients are very aware of this problem and understand that, without their active participation, the situation for them, their genetic relatives, and all other people will have never improved. On request for their agreement, many are ready to provide their medical data and even samples of body fluids or tissue for future research use (Brull et al. 2004; Moorcraft et al. 2016). Of course, the hope is that they will receive improved medical interventions in return. It is critical that such benefits, as distant as they might be in time, are actually delivered when such an opportunity arises and that mechanisms exist to support that option. Among other aspects, this benefit requires that patients can potentially be contacted also after immediate research efforts have ended. Thus, patient data sets need to be labeled with permanent anonymized patient identifiers that remain resolvable for actual patient names and contact data at any time point after the study.

The probability of individual advantage from joining a trial is small; nevertheless, patients do participate and accept that progress in medical science might take a long time and that they or their living relatives might not directly benefit from their personal generosity during their lifetime. Such an outcome is generally still tolerated as long as society at large will be the main beneficiary of their donation of data and samples. To create this atmosphere of trust, more than just a legal framework and an ethical oversight body are necessary but also social protection mechanisms, societally supported health insurance instruments that shield people in extreme cases, etc. As non-mainstream life situations, unfortunate medical conditions such as psychiatric diseases, etc. will surface in the process of medical data studies, the issues of preventing social stigma and discrimination (for example in job and insurance matters) become critical. So, the tolerance and empathy level in society will be a factor that can enhance or inhibit further progress. It appears to us that, possibly, new mechanisms need to be developed to acknowledge people donating data for research, such as participating in economic success stories that ultimately have been achieved as a result of studying these data.

Carefully selected, meaningful benefits given to patients that allow using their data would also play a positive role in the consent process. Patients could be offered to be serviced by research hospitals where diagnostics and treatment are promised to be at the edge of medical science and, to some extent, costs are subsidized in exchange that all the patient data collected will be unrestrictedly used for medical research, now and in the future.

## Access to Medical Patient Data in Biomedical Research: the Patients’ Views with Respect to Consent

Thus, to receive the consent from patients to release their data for various purposes of medical research is not the greatest difficulty for scientists to get access to them at present when certain conditions are met, as their interest in better medical science is self-evident. Yet, it requires sensitivity and considerable effort to gain the patients’ support and agreement and, in parallel, to fulfill the legal requirements.

The current practices in requesting and documenting the consent of patients are not very suitable for supporting large-scale medical research on patient data. Leaving the full burden and risk of the patients’ consent on the shoulder of researchers and their institutions who have very limited resources is not an arrangement that will widen the scale of medical data research, something that is not in the society’s best larger interest. Thus, some societal support is needed to make clinical data research possible and a standardization of consent documentation and procedures would help. A standardized form for broad consent or at least a template that is approved by government legal authorities, top courts, or other relevant legal bodies such as the Institutional Review Boards bestowed with the mandate to review clinical research in Singapore would be helpful in this process. We recognize that consent is a process informing patients about the issue of using the data for research and getting them agreeing. It is not just an act of signing a document. Yet, a consent form that could be routinely used in the process and that makes sure that the legal conditions are met provides a useful shortcut saving time and money.

Often, consent is asked for a single clinical trial and the data collected cannot be legally used for other scientific purposes. This is a waste of economic resources as especially omics and image data collected for one study might be useful (even if not fully optimal) for a completely unrelated research that was possibly not obvious at the time of collecting the data. An alternative would be to request much broader consent that allows storing the collected data in anonymized form in clinical databases for all kinds of medical research including future efforts (Coughlin 2011; Faden et al. 2013; Karlsen et al. 2011; Sheehan 2011a, b).

## Access to Medical Patient Data in Biomedical Research: the Patients’ Views with Respect to Data Anonymization

Finally, there is the unresolved issue of anonymization of medical data. It is well known that just removal of names, addresses, and phone numbers from medical records does not always prevent establishing the identity of the patient from the remaining data (Brenner 2013; Gymrek et al. 2013; Harmanci and Gerstein 2016; Na et al. 2018; Shringarpure and Bustamante 2015). For example, patient identification can be possible due to private information embedded in medical images such as X-rays or MRIs that is difficult to remove automatically without damaging the image itself. Various pieces of anamnesis data taken together might suffice to discover the patient’s identity, especially if linked with other databases, for example diverse public data repositories, telephone books, social media such as Facebook, etc. The introduction of omics data finally compromises the concept of anonymization as the genome sequence and all derived omics data are ultimately individual and are always sufficient to find the patient in a cohort. To emphasize, there is no anonymization with omics data as every genome signature is personal by definition.

Thus, the currently widely accepted solution is to make patient identification from so-called anonymized data difficult by removing the obvious pointers, to condemn data misuse morally, and to prosecute it legally. Several additional technical layers are either in place or being explored for restricting data access by non-eligible parties and to prevent unauthorized ways of analyzing the data. First, medical patient data is typically stored in especially protected, ISO27001-certified, strict access-controlled datacenters:

(i) with enhanced technical system reliability that ensures data longevity and application survival even under detrimental conditions (including a backup and business continuity strategy),
(ii) with automated documentation of life time activity over data and applications, and
(iii) with physical and electronic access protection, security control and log protocol. Only an explicit list of eligible users is allowed to access predefined portions of the data, their analysis activities are automatically watched and documented.

Several further privacy-enhancing measures are being considered and explored at present (Cho et al. 2018):

(i) Block-chain-type distributed ledgers might be implemented to register data deposition and usage (Wong et al. 2019). It might be problematic to put a ledger to every elementary piece of data as the computational overhead can become prohibitive but, for example, applying them only to larger blocks such as the patient entry as a whole could be a compromise solution.
(ii) There are sensitivities of organizations and nation states that do not allow their patient data to leave the limits of their territory. Sometimes, this matter could even make an issue of national sovereignty. Besides ethical concerns, also economic considerations of data owners play a role here. In such cases, there is no way around executing computations, queries and analyses, over federated, locally dispersed databases without downloading the data centrally. In Singapore, the software CHORUS was developed for this purpose (Ng 2016).
(iii) The ultimate protection of privacy might be achieved by encrypting the patient data and performing analysis on encrypted data without ever revealing the data explicitly to the analyzing scientists (Jagadeesh et al. 2017). This approach is possible if the way of analyzing the data has become increasingly routine (e.g., as requesting the identity of nucleotide bases at certain positions in GWAS studies) and data processing methodology applied does not require lots of contextual inputs.

All of these solutions drive up overheads dramatically and make the research costlier. Further, they limit the number and diversity of scientists that can participate (thus, shrink the amount of creativity applied to the problems). The diversity of analysis approaches and methodologies gets limited as every change will require new software adaptations that have to be approved by audit. Even the basic principles of scientific work such as manual assessment of outliers in the context of other data are no longer applicable in such environments. Thus, overemphasizing the privacy aspect of patient rights in medical data handling is actually damaging to the more basic patient interest of having dynamic progress in medical science.

The authors of this article think that block-chain ledgers, federated databases, and encrypted computation will have their role in medical patient data studies when simply no other access to these data is possible. Yet, we are convinced that the principal advances in this field of research will be made with datasets provided with more lenient access rules since data gazing, creative experimentation with various data items, and community-wide exchanges play a major role in scientific advance. Encrypted, federated databases become useful when principal aspects of the methodology are established and more accurate computations due to the larger data size are warranted.

## Access to Medical Patient Data in Biomedical Research: the Physicians’ Views

There is not only the patient interests’ side. The public discussion usually forgets that collecting medical observation data for studies is extremely expensive and one of the most costly modes of life science research (Collier 2009; Martin et al. 2017). For example, a typical single clinical trial covering all clinical phases runs into ~ 50 million USD (Sertkaya et al. 2016). This is comparable to the accumulated costs of a large biological scientific research institute with ~ 250 researchers per year that produces at least 100 substantial research papers per year. Consequently, given the great resource expenditure, it appears necessary to extract the maximum benefit for society out of trial data, regardless whether the money invested is public or private. Thus, publicly funded clinical trials should mandatorily make their data a public resource, something that does not happen routinely today. And it is desirable to provide public incentives to private investors to supply their clinical trial data to research depositories once they have exhausted their own analysis effort.

The large investment of working time and mental capacity by clinical staff is one of the main reasons for the cost of collecting clinical data. Thus, there is great sensitivity and justifiable interest from the physicians’ side to extract at least some personal recognition for their investment in data collection and to secure this goal by limiting the access of competing researchers to “their” data. This circumstance has several critical implications:

(i) As the physicians are also the only ones in the data analysis chain who directly interact with the patients, they become the guardians of and the gateway to the patients’ data. From the practical point of view, this position leads the clinicians becoming the factual owners of the patients’ data, an additional role that actually conflicts with but also shadows their vested interest as career researchers.
(ii) Due to their specific professional background with, naturally, limited exposure in information technology, formalized data analysis and complex, sophisticated approaches in computational biology and bioinformatics, clinicians are not the optimal drivers for certain aspects of the data analysis process. For example, it happens in practice that the data collected in a clinical trial actually resides in disjunct pieces on thumb drives in the hands of participating doctors without any protection of data loss and authenticity. Omics and imaging data analysis requires sophisticated specialized abilities. Hence, collaborators, professionals in medical data handling and analysis (we call them bioinformaticians in this article) are needed that have to be given access to the data at an early stage before most value from the data could be skimmed off.

The conflicting interests described above between physicians and non-clinical researchers (many of whom are also medically educated and trained) in a project were not so important previously as the part of organizing and interpreting the data was not that essential to clinical research in previous decades. In an extreme case, the clinical party might assume that their factual ownership of the data implies ownership of all medically relevant conclusions and that the non-clinical researchers provide only a technical service to them without creative contributions. This assessment certainly does not reflect the reality and will also not stimulate the other party to fully invest itself in the analysis effort. At the same time, bioinformaticians might forget at the height of excitement in the moment of discovery that it was the clinical collaborators who enabled them to see the conclusions because of the systematically collected medical data. Both extremes are detrimental for a successful collaboration.

Thus, it becomes critical to achieve the rapport of the clinical researchers in the medical data analysis process, to dutifully acknowledge their contribution in data collection and its primary quality control and data analysis. This might be difficult to achieve since the time between the data’s collection campaign and the moment of extracting a useful insight from them might be considerable involving many years or even decades in the extreme. That means that all clinical datasets need to be accompanied by names and contact details of the clinicians involved so that they can be informed about possible research outcomes and that their clinical comments and their personal agreement can be requested before exploiting their insights for publications and other purposes and before giving the information to the public. Currently, the procedures for exploiting long-term clinical databases for research and requesting consent from the physicians are not well elaborated once the data collection phase is in the distant past and contacting some of them might be problematic as they have moved on. Standardized protocols or executable agreements would be desirable in this context.

## Access to Medical Patient Data in Biomedical Research: the Bioinformaticians’ Views

Whereas clinical data analysis might have only involved cursory biostatistical expertise in addition to the clinical contribution a few decades ago, the large amount, the heterogeneity, and complexity of medical data, especially in cases with omics and certain imaging components, require additional input from professionals in the area of information technology and computational biology/bioinformatics. In the process of transforming medical data into actionable medical insight, the bioinformaticians’ group contributes two important functions, namely (i) the creation of electronically analyzable datasets and (ii) the analysis and interpretation of this data (in collaboration with the clinical partners). The group’s interest is driven by the possibility of and the professional recognition due to scientific discovery and its subsequent bedside application.

It is very important to correctly understand the role of the biomedical data scientists in the process of medical data analysis. By curating, annotating, and cleansing patient datasets, a sophisticated service is provided to the research community, especially to the clinical partners. The data janitors’ work as described further below is an independent, valuable effort with both sophisticated routine and creative elements. The creation of error-free, long-living databases for medical patient data are necessary for reproducible biomedical research and reliable scientific conclusions; thus, it is an ethical imperative on its own (besides its economic importance) as erroneous data will eventually support useless, wrong or even detrimental clinical conclusions. This happened many times in the history of medicine, for example in the case falsely linking autism and vaccinations (Hoffman et al. 2019; Hviid et al. 2019).

The actual data analysis work should be seen as additional and independent of this database creation effort. The wider the access to the medical data is among the scientific community and the more minds (including non-clinicians) lend their attention to the analysis of the data, the higher is the likelihood of discovery. Referable databases are a precondition for such a scientific endeavor to happen. Thus, it is at this stage where ethical issues of handling medical data as well as information-technological and biomedical science aspects get most closely intertwined, where well-minded, overprotective ethically defended regulations will botch the opportunity of innovation that are so desired by the patients.

For achieving a workable compromise between stakeholders, medical data ownership once the records have entered electronic databases for research needs to be clearly demarcated. To note, the bioinformatics side does not become the owner of the data; rather, it is the guardian of its physical quality, the technical access, and the scientific usefulness. It provides a form of convenient usage to the data. For the various clinical contributors involved, it is a neutral party standing at the side of their potential rivalries and competitions serving everybody equally. Therefore, it should be freed from the ultimate function of decision making about allowing access to the data or about the exploitation of study outcomes. This is rather to be delegated to the governing body of the research consortium where the bioinformatics contributors in addition to patients and clinicians also should have a voice.

As the studies conducted are typically multi-centered with many parties contributing for clinical data collection and analysis, the apparently mundane issues of data collation and alignment, standardization, release versioning, long-term data storage and data access provision to consortium members and possibly other eligible data analysis parties become critical for the success of the whole effort. On the one hand, it is important to maintain the body of data for the longer term (data longevity), during the trial or diagnostics/treatment cycle as well as many years beyond the immediate work. On the other hand, specific data analysis studies need to be linked with temporarily defined snapshots of the datasets (release versions) so that conclusions can be reproducibly linked with specific clinical data points. Finally, the data collation should enable cross-sectional analyses over heterogeneous datasets collected by the various clinical parties involved.

## Access to Medical Patient Data in Biomedical Research: Experiences at the Bioinformatics Institute Singapore (BII)

At BII, we have gone through the process of data curation for a number of clinical trials with hospitals, diverse academic, and industrial partners. Below, we share our generalized experiences. Data collation from various consortium partners in a multi-centric effort is not a trivial process, and it involves stages of alignment, data cleansing, and dataset versioning. In particular, the scale of the necessary data janitor effort surprised us.

With the processing pipelines implemented as part of our own in-house developed database software TIMS (Translational Information Management System), the records uploaded by the clinical partners will be aligned along patient codes so that different data from one and the same patient can be jointly analyzed. Image and omics data are processed from their raw form into formats that can be analyzed at later stages. Upon data upload, an evolving suite of quality checking routines is applied. Depending on the type of data, we check completeness of records, data error margins, the likelihood of the data points being really collected from the same patient (for example, via plausible levels of sequence identity in various datasets involving DNA/RNA sequencing, mismatches in medical images, etc.), consistency of various groups of data items (e.g., gender- and age-specific trends in laboratory data, physiological limits), etc. If inconsistencies cannot be resolved automatically, request lists are generated for our clinical partners to review. To note, our clinical partners were first worried about the large feedback about data inconsistencies. They were embarrassed and feared that they would appear non-professional but, later, they were happy to see that the quality of the datasets and the statistical power of conclusions improved substantially after this janitorial work. Below, we will detail about some of the observations we have made in uploaded medical records.

Only when no issue remains, the patient records are released for further analysis. It is thought that, after the data cleaning procedure, there will be no more obvious inconsistencies, non-acknowledged missing data points, and data with error margins above reasonably defined thresholds. Data quality assessment protocols are stored alongside with the data itself by TIMS.

The data is provided to the consortium members and other eligible parties in a collated and curated manner. Time-stamped data release versions are generated so that all conclusions drawn can be unambiguously linked with a defined release of the dataset and computations could be repeated if desired. For data security, medical data analyses at the Bioinformatics Institute Singapore are always performed on copies, never on the master database. The master database contains the raw-data, cleaned data and time-stamped processed data which is located at the high-sensitivity zone within BII ISO-27001 certified network. The time-stamped processed data can then be exported to low-sensitivity zone for data visualization among eligible members. For an independent data analysis of the processed data, data access is provided in three ways. (i) Eligible users might wish to copy segments of the data, transport/transfer them to their premises and carry out the analyses there. (ii) In cases of small data pieces needed from many patient records, WWW-based Internet access can be provided via visualization engines such as cBioPortal (Gao et al. 2013). (iii) Finally, eligible users will be provided with the opportunity to carry out the analyses within the premises of the Bioinformatics Institute using their or our software tools. As datasets can become very large, this is the most convenient way.

## Usefulness of Patient Datasets for Biomedical Research: What Can Go Wrong in Patients’ Records Does Indeed Go Wrong Sometimes and This Has Consequences for the Conclusions

The practical and ethical implications of inaccuracies and errors in medical records are rarely discussed in the scientific literature and, in the public, their scale and even mere existence is largely unknown. When developing our software pipelines for clinical data processing, we had brainstormed about possible inconsistency checks in patients’ records, implemented these ideas into programs and we were surprised to see that, with enough incoming data, instances for every such error were indeed found and, in some cases, their frequency would have led astray data analysis using the raw, uncorrected data. Below, we will present a couple of examples.

As patients visit physicians at diverse facilities over longer periods of time, certain data types such as anamnesis or laboratory data are repeatedly collected and these time series should be expected to be consistent. For example, gender or ethnicity (in the Singapore context, there are three major ethnicities living in the city: Chinese, Malays and Indians) are inherent properties of an individual and, typically, they should not change over time. Nevertheless, we noticed cases that Chinese individuals were sometimes labeled Indian or Malay or vice versa. Such mix-ups are ethically unacceptable. It represents a sensitive matter as there are ethno-specific aspects of physiology and culture influenced by life style, religion, etc.

Similarly, we found cases of male and female codes coexisting in different parts of the data for one and the same patient without gender change being confirmed by the responsible physician. Even worse, male and female encoding was completely interchanged for all patient records in one type of laboratory study of a clinical trial. If this subset of data were analyzed independently, the conclusions would have been wrongly assigned to the opposite gender. We also found quite some fluctuations in physical measurements such as height. Differences between records for the same patient made at different hospitals can make up to 20 cm. We also check whether date labels of records for one and the same patient are consistent with the medical information provided. For example, one would not expect certain measurements of vital signs being recorded after death; yet, we did see such cases.

**Fig. 1:**
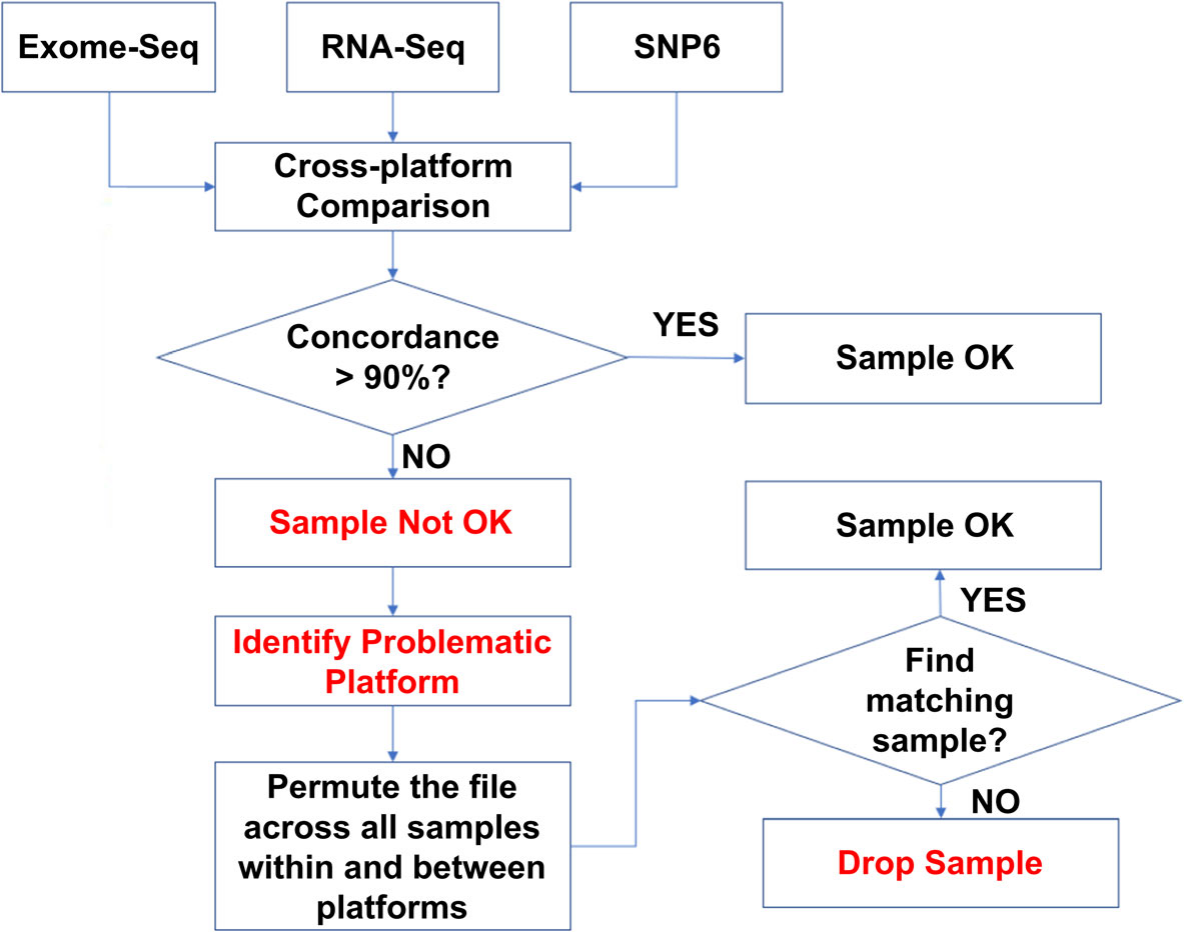
Omics data consistency checking algorithm. The genome signature is personal and all omics data from one and the same patient should be alignable to the same genome matrix be genome sequence, transcription profiles, oligonucleotide, or protein array data. We have a concordance check workflow for multiple omics dataset in place. Sample mislabeling is identified following high discordance across platforms. Mislabeled samples can be automatically fixed following permutation across all other samples if the correct patient is part of the cohort

As the individual genome sequence leaves a signature in all types of omics data, it can be established very reliably whether all omics datasets with the same patient label are actually from the same patient (or from another patient in the same database or even from yet another person). We have developed our own in-house workflow for quality control of patients’ label when multiple omics datasets of the same patients exist (Fig. 1). Briefly, multi-omics data from the same patients are evaluated for the concordance of their genomic footprint if available. Patients with high discordance in their genomic information across different platforms will be permuted across all patients’ omics data and identify the potential matching. As it is obvious that random pairing of patients’ omics data has low concordance, therefore any pairing with high concordance should reflect the patients matching. We were surprised to learn that, in a cohort with three omics datasets per patient (DNA-seq, RNA-seq and SNP array data), we found about 10% of patients having at least one sample being mislabeled. This data cleansing was critical for this study. For the original data, there was not a single gene expression signature that was a statistically significant classifier of two groups of patients. Yet, the corrected datasets clearly allowed delineation of a group of biomarker genes with great predictive power.

To note, profane matters such as missing data, typos, and formatting inconsistencies are much more frequently observed. Differences in orders of magnitude for laboratory values usually indicate that measurements have been recorded in various units (e.g., cm or m for height, ng/ml or ng/l for plasma protein components, etc.). For the human reader, a string or a numerical value is equally read. For a computerized routine, this makes a big difference.

Of course, none of these sometimes funny inconsistencies are intentional. We think that many errors in clinical records are the result of tired nurses and doctors having to process large amounts of patients. We think that substantial numbers of these problems could be avoided by intelligent input masks that, without suggesting some standard values, could make background checks and inform nurse or doctor about the inconsistency at the time of input. Further, measurements could enter records automatically from devices.

When looking into the biomedical literature, we were surprised to find quite scarce reporting about clinical data cleansing (Beaulieu-Jones et al. 2018; Chen et al. 2018; Coiera et al. 2016; Ehrenstein et al. 2017). It appears to us that not all in the community are aware of the scale of the problems. We think that the success and/or failure of at least some of the clinical trials in the past might be attributed to the errors in the body of data, especially in cases of marginal statistical significance of conclusions that are not stable upon randomization of a small subset of the data.

## Usefulness of Patient Datasets for Biomedical Research: Clinical Trials, Electronic Health Records, Abbreviated Medical Record Sets and the Importance of Billing Information

So far, the authors of this paper have worked primarily with clinical trial data. The clinical partners invest enormous efforts to collect good patient cohorts that lead to long time series of data recorded in a variety of modalities. As a result of a joint effort, high-value datasets free from obvious inconsistencies and incompleteness are generated that, as we hope, will be of value for biomedical research in Singapore for many years to come.

Compared with the clinical trials, EHRs collected as part of the routine clinical practice in hospitals and clinics are much larger with several orders of magnitude more patients. Clearly, this opens additional opportunities for finding patterns (Jensen et al. 2014). Nevertheless, we think that research work on EHRs in the foreseeable future cannot compete with the outcome achieved from well-curated clinical trial data analysis due to data quality issues. This problem has even been highlighted in the general press (Tan 2017). For example, 27% of patients’ entries in Scotland’s national electronic health registry are thought to contain errors and almost half of those errors are considered clinically important such as diagnoses. The author also mentioned the discrepancy between the working time needed for documentation and evaluation of all data in a patient’s EHRs on the one hand and overload and the long work hours for clinicians that cause them risking burnout on the other.

In addition to all the factors discussed in the previous section that deplete the value of clinical trial datasets, further factors come into force for EHRs. So, it is practically impossible to organize feedback from hundreds of doctors to review cases of inconsistency in their patients’ records, especially if these records have been made some time ago and the patient might be no longer available. Further, aspects of reimbursement by insurances frame the style of diagnosis and reporting in the EHRs. For example, when the fee is calculated by the list of ICD (International Classification of Diseases) codes, there is a trend for not very clearly defined diagnoses such as hypertonia to proliferate among the records.

The groups of computational biologists, bioinformaticians, and biostatisticians that work with clinicians over clinical trial data belong to a very privileged but small group of scientists as they have access to clinical data of their patient cohort in their entirety. The whole breadth of scientific work principles such as application of non-standard analysis approaches, the gazing over outlier data and the comparison of thought outcomes from studying various clinical diagnosis modalities and time series is possible.

In contrast, the majority of scientists have no or little access to clinical data when they are not members of consortia with clinical partners and their creativity and ingenuity will not be applicable to such scientific problems. Further, ethical review of research plans, especially if applied restrictively, can further shrink the circle of eligible researchers. Some might simply avoid the hassle of yet another bureaucratic procedure, especially if clinical data study is not at the core of the project. So, the competition in this field of science gets severely restricted.

Recently, some publicly available repositories provide access to clinical data in a quite abbreviated form. There are a few public datasets available that offer collections of records with an omics or imaging modality data associated with diagnosis, disease outcome (e.g., survival status) and minimal anamnesis data such as age and gender. For example, in oncology, these datasets come usually as collections of genomes or RNA-seq transcription profiles for patients with similar tumors. The Cancer Genome Atlas (TCGA) is one of the most advanced examples (Wang et al. 2016). In other cases, human data is contained in the database aside with data from other organisms as in the Gene Expression Omnibus (GEO) (Barrett et al. 2013).

Usually, there are no complete medical history, repeated analyses, or an alternative modality available. It is not possible to carry out internal consistency checks over these datasets to the extent as we learned to do with clinical trial data. To note, annotation errors introduced by the data provider (such as unintentionally but systematically mislabeling gender or ethnicity as we mentioned above) can dramatically compromise the value of the analyses’ outcome.

It should be noted that almost all of the scientific studies with patient data work with records where billing data is stripped off. In our eyes, this needs reconsideration in the future as absence of pricing information will misdirect human efforts, also in the scientific sphere. To note, there is no free lunch, especially not in the healthcare sector. Exact costs for diagnosis and treatment are generally unknown among patients and scientists, especially in environments of full reimbursement by national insurers. In the absence of knowledge about the economic impact, scientists might dedicate lots of effort in refining diagnosis and treatment of certain conditions; yet, introducing these improvements into practice will have little impact for individual patients and for the system as a whole. Increasing sensitivity to economic aspects will lead to more reasonable suggestions and research tasks at the research side and better societal outcomes of the research investment.

## Discussion and Conclusions

The question of access to patient data for biomedical research is widely seen only from the angle of patient privacy. Whether this is due to simplicity or hypocrisy does not matter. The outcome, namely that the majority of biomedical researchers has no or very abbreviated access to clinical data, is not good for the patients as the extraction of valuable insight with impact for diagnosis and treatment is delayed or hindered at all. To note, bioethical research needs to ask itself to what extent ethical considerations and their legal implementations are no longer protecting the actual patients’ interests but rather become tools to suffocate research initiatives and to protect vested interests of certain groups with the outcome of delayed or no innovation. Either of the extremes is not ethical. Yet, the current approaches rather suppress innovation that could be fueled by allowing analysis of costly omics and bioimaging data associated with clinical data by as many as possible teams in the research community. In addition, medical data protection is bought with a costly administrative overhead and, so far, there is no answer concerning when this expense has reached a level that is no longer justifiable ethically, for example when even small research units need to employ personnel exclusively to comply with regulations and audit.

Without doubt, the patients’ data privacy right is an extremely valuable good. But the right to live and to live without disability has a much higher value in the eyes of patients. At the same time, it appears that currently, as exemplified by the 2018 cyberattack on SingHealth, a major health care provider in Singapore, hackers have easier access to patient data than researchers (Tham et al. 2018) when the hackers have no intentions to improve biomedical insight or healthcare. To note, medical data for about 1.6 million Singaporeans (one third of the population) have been downloaded into data storages at the dark net in this attack. Therefore, instead of trying to install absolute protection for patient privacies, it seems rather more realistic to think about insurance mechanisms that could kick in once patient data gets leaked and people’s lives get damaged as a result of it.

So, it is generally not the patients who create the major obstacle for the patients’ data reaching biomedical researchers and it is hypocritical and simplistic to represent them as the core of the problem. Scientific work is done by human beings and, in addition to patient interests, needs of other stakeholders have to be considered. Medical data collection by clinicians and health care institutions is a time- and cost-intensive process. It is most of all tedious and exhausting, with few elements of intellectual and emotional satisfaction on its own. The contribution of the medical staff involved in this process cannot be underestimated and, as we are convinced, the technical support that they receive to manage the enormous workload is insufficient. Computer intelligence-fueled input masks, automated transfer of laboratory data into patient files, automated consistency checks in the backgrounds combined with specifically generated questions to the doctor or nurse involved in the data input, etc., are just few of many improvements that are required.

Patient datasets as they come from the clinical side are not very useful for research, as they contain lots of errors and inconsistencies. It is important to introduce another layer of data “janitors” to eradicate as many as possible problems with the data before any serious analysis. Then, time-stamped version of the data should be provided to the various data analysis parties so that the studies become reproducible.

The issue of reusing clinical trial data both after and beyond the originally intended study has not received sufficient attention in the community. We think that clinical trial data should enter dedicated databases with long-term storage to allow future re-analysis as well as cross-trial studies of various kinds. Without repeating the major investment for data collection, many more insights can be extracted from the same data given the more sophisticated analysis possible at a later time point. Thus, additional incentives and mechanisms for protecting interests of respective stakeholders are necessary in the process. Publicly financed clinical trials should be first in making their data widely available to research including the non-clinical communities.

At the biomedical researchers’ or even principal investigators’ level, it is very difficult to change the macro-environment that would allow a more generous access to medical data when, outside of the biomedical research, this is not even reflected as an acute problem. Perhaps, the bioethics community might join the fight by asking to which extent the potential loss of innovation opportunity or the increased research cost by a new principle or law (for example, with new the Human Biological Resource Act (Ministry of Health Singapore 2019)) is ethically justified. Formalizing the broad patient consent for clinical trials or for admission into high-tier hospitals with strong research divisions would have a lasting impact as it will slowly create a body of data and biological samples that can be re-analyzed in many types of research contexts and by a wider group of researchers. There is an ongoing discussion about ‘learning health systems’ (USDHHS 2019) that need to reuse their own data for improved and more efficient services. Even the otherwise quite restrictive European Data Protection Regulation (EDPR) contains a general exemption for data processing for research in the public interest where consent of the subject (except at the initial anonymization procedure) is not required (European Union 2019). Hopefully, the awareness for the problem of slowing down and even stalled innovation in biomedical research will gradually increase (Sinha et al. 2018).
